# Measurement and stratification of nonsuicidal self-injury in adolescents

**DOI:** 10.1186/s12888-024-05535-3

**Published:** 2024-02-07

**Authors:** Erik Aspeqvist, Hedvig Andersson, Laura Korhonen, Örjan Dahlström, Maria Zetterqvist

**Affiliations:** 1 Center for Social and Affective Neuroscience, Department of Biomedical and Clinical Sciences, Linköping University, Linköping, Sweden; 2 Department of Child and Adolescent Psychiatry, Region Östergötland, Linköping, Sweden; 3 Barnafrid, Swedish National Center on Violence Against Children, Linköping University, Linköping, Sweden; 4 Department of Behavioural Sciences and Learning, Linköping University, Linköping, Sweden; 5 Athletics Research Center, Linköping University, Linköping, Sweden

**Keywords:** Nonsuicidal self-injury, Adolescence, Community sample, Measurement, Cluster analysis

## Abstract

**Background:**

Nonsuicidal self-injury (NSSI) is highly prevalent in adolescents. In survey and interview studies assessing NSSI, methods of assessment have been shown to influence prevalence estimates. However, knowledge of which groups of adolescents that are identified with different measurement methods is lacking, and the characteristics of identified groups are yet to be investigated. Further, only a handful of studies have been carried out using exploratory methods to identify subgroups among adolescents with NSSI.

**Methods:**

The performance of two prevalence measures (single-item vs. behavioral checklist) in the same cross-sectional community sample (*n* = 266, age *M* = 14.21, 58.3% female) of adolescents was compared regarding prevalence estimates and also characterization of the identified groups with lifetime NSSI prevalence. A cluster analysis was carried out in the same sample. Identified clusters were compared to the two groups defined using the prevalence measures.

**Results:**

A total of 118 (44.4%) participants acknowledged having engaged in NSSI at least once. Of these, a group of 55 (20.7%) adolescents confirmed NSSI on a single item and 63 (23.7%) adolescents confirmed NSSI only on a behavioral checklist, while denying NSSI on the single item. Groups differed significantly, with the single-item group being more severely affected and having higher mean scores on difficulties in emotion regulation, self-criticism, number of methods, higher frequency of NSSI, higher rates of suicidal ideation and suicidal behavior and lower mean score on health-related quality of life. All cases with higher severity were not identified by the single-item question. Cluster analysis identified three clusters, two of which fit well with the groups identified by single-item and behavioral checklist measures.

**Conclusions:**

When investigating NSSI prevalence in adolescents, findings are influenced by the researchers’ choice of measures. The present study provides some directions toward what kind of influence to expect given the type of measure used, both with regards to the size of the identified group and its composition. Implications for future research as well as clinical and preventive work are discussed.

**Supplementary Information:**

The online version contains supplementary material available at 10.1186/s12888-024-05535-3.

## Background

Nonsuicidal self-injury (NSSI), defined as the intentional, direct destruction of one’s own bodily tissue without suicidal intent for purposes not culturally sanctioned [1], is common in many countries [2], and especially in the adolescent population [3, 4]. NSSI is generally understood as a functional behavior employed as a coping strategy [5] and thus is not in itself an expression of suicidality. It is, however, statistically associated with suicide attempts [6] and with psychiatric disorders, such as borderline personality disorder, post-traumatic stress disorder and depression, especially in clinical samples [7–9].

During the last 10–15 years, the research field of NSSI has expanded, and considerable advances gave been made in the field of epidemiology and characteristics of NSSI, including prevalence rates, gender differences, age of onset and prognosis [10]. Measuring NSSI prevalence and characteristics in the adolescent population has not been without challenges, however. Despite the progress, there are still issues that need to be addressed and clarified, some of which are outlined below.

### Characteristics of NSSI in adolescents

The mean age of NSSI onset is around early adolescence, 12–14 years [7, 11, 12], but NSSI can begin as early as age seven [13]. Rates of NSSI typically peak at around 15–16 years, after which the overall pattern is that rates of NSSI drop with increasing age [14].

Results indicating that NSSI is associated with both emotion regulation and self-critical cognitions or self-punishment desires have contributed toward the understanding of the mechanisms behind the behavior [5, 15–17]. Emotion regulation has previously been identified as the most commonly expected function of NSSI behaviors [5], and the introduction of the NSSI expectancy questionnaire (NEQ) [18] has made further study of cognitions related to NSSI possible. The results of the few studies employing NEQ that have been carried out so far generally show that respondents with NSSI expect emotion regulation effects to a larger degree than those without NSSI, and to a smaller degree expect communication effects and experiencing pain [19–21]. It should be noted, however, that earlier studies have used adult samples of students. To our knowledge, NSSI expectations have not been investigated in child or adolescent samples.

Systematic reviews have established that up to half of adolescents with experience of NSSI do not seek help, and that the ones who do seek help often turn to friends and family rather than to professionals [22]. Adolescents’ cognitions, including expecting shame, fear of negative reactions or of being judged or misunderstood, are barriers to disclosure and help-seeking [23]. Common misunderstandings among young people in general include the idea that only girls self-injure, that NSSI is an indication of psychiatric disorders, as well as a misconception of NSSI as a manipulative behavior [24]. For these reasons, knowledge of how young adolescents view NSSI, and perceive issues such as stigma and help-seeking, is crucial when working with treatment and prevention.

Regarding NSSI methods in adolescents, cutting and scratching have been found to be more common in females, while males are relatively more likely to use burning, banging or punching [25]. Attempts have also been made to grade the severity of NSSI methods, placing some methods (e.g., cutting/carving, burning) in a moderate/severe category and others (e.g., hitting self, biting) in a minor NSSI category [26]. Further, a larger number of methods used to self-injure, higher NSSI *versatility*, has been associated with greater risk of suicide attempts [27–29] and thus might be cautiously used as a proxy for NSSI severity.

### Measurement of NSSI prevalence

Determining the extent to which NSSI occurs in different populations has been and continues to be an important task for the research community [10]. In the past decade, two well-cited reviews placed lifetime prevalence rates of NSSI in community adolescents at 17–18% [2, 30] and a more recent meta-analysis resulted in an estimation of 22% [31].

The heterogeneity of prevalence findings has, however, been striking. Among the studies included in the meta-analysis by Xiao et al. [31], lifetime prevalence rates in community samples of adolescents ranged from 4.3 to 47.1%. Muehlenkamp et al. [2] highlighted the heterogeneity in measurement methodology as an important contributor to the discrepancies between prevalence estimates. Swannell and colleagues [30] followed up on these findings and, among the studies included in their review, identified 76 different tools used for measurement of NSSI, including structured and semi-structured interviews, as well as shorter and more expansive self-report questionnaires.

How NSSI is measured varies across both self-report and interview measurements. Methods include on the one hand, measuring with a single dichotomous question whether the participant ever engaged in NSSI and, on the other, providing a list of NSSI behaviors, using a so-called behavioral checklist. Commonly used instruments including NSSI behavioral checklists are the Functional Assessment of Self-Mutilation (FASM) [32], the Deliberate Self-Harm Inventory (DSHI) [33] and the Inventory of Statements about Self-Injury (ISAS) [34]. The Self-Injurious Thoughts and Behaviors Interview (SITBI) [35] includes a single question as well as a behavioral checklist. The checklist is, however, only administered if the participants answer positively on the single question. Building on the finding by Muehlenkamp et al. [2], that behavioral checklists resulted in prevalence rates that were almost doubled compared to single-item questions, Swannell and colleagues [30] computed the contribution of methodological factors to the heterogeneity of NSSI prevalence estimates. They found it to be 51.6% and concluded that variability in prevalence estimates is “heavily influenced by features of the measurement tool, especially when the research tool is a checklist rather than a single yes or no question” (p. 280). A difference in prevalence rate was also found in the same study sample [36], with prevalence rates of 35.6% and 17.2% in a community sample of adolescents with checklist and single-item, respectively. Aiming to explore these discrepancies further, Lund and colleagues [37] compared a single-item question to two behavioral checklist measures: DSHI [33] and ISAS [34]. Previous findings that behavioral checklists yield higher prevalence estimates were repeated, and the authors could also show that the group that indicated NSSI on the single-item question differed in that its members reported significantly higher numbers of different NSSI methods (methods used when engaging in NSSI, including but not limited to cutting, scratching or hitting oneself) [26], higher NSSI frequency, as well as greater psychological distress than the group that only indicated NSSI on a behavioral checklist.

Robinson and Wilson [38] investigated some of the explanations put forward regarding the discrepant performance of single-item and behavioral checklist measures. The authors demonstrated that neither careless responding nor memory facilitation by the behavioral checklist could explain the differences; the order in which the measures were presented did not have any effect on the participants’ responses. They also pointed out that as some survey and interview measures use a single item for branching (“if no, skip to next section”), participants who do not indicate NSSI history on a single-item but still acknowledge NSSI on a behavioral checklist, would be incorrectly screened out. Robinson and Wilson [38] further provided descriptives for the screened out group, and replicated the findings of Lund et al. [37] that fewer participants indicate NSSI on a single-item. The group indicating NSSI on a single-item also reported a larger number of NSSI methods than the group indicating NSSI only on a behavioral checklist. Differences were also shown between NSSI methods, where cutting and burning were associated with indicating NSSI on a single-item. Further, more male participants were found in the group reporting NSSI only on a behavioral checklist. Robinson and Wilson [38] concluded that “the common practices of measuring NSSI with single-item assessments or two-step procedures are more consistently capturing people who self-injure by cutting, but is likely missing people who engage in less prototypical behaviors or those who do not identify as someone who self-injures” (p. 734). They suggested that moving forward, research could focus on describing the groups captured by the different assessments.

Both the study by Lund et al. [37] and Robinson and Wilson [38] used adult samples and only explored group differences on NSSI methods, psychological distress, and demographic data. Therefore, more information from adolescent samples is still needed on which groups are identified with different measures, as well as further details on how these groups differ on other variables.

### Different groups of individuals with NSSI

In the research community, consensus is yet to be reached regarding which criteria would be meaningful to identify which behaviors to consider as acts of NSSI, as well as to differentiate individuals within the group with a history of NSSI [39, 40]. How the risk of severe and/or prolonged NSSI is distributed is clinically valuable information, but only a handful studies in this area have been carried out so far. To provide examples of the approaches suggested to identify NSSI subgroups, we present a selection of such studies below, divided into those with top-down and bottom-up approach.

#### Top-down approaches

Brunner and colleagues [41], for example, investigated group differences among adolescents with occasional (up to three yearly episodes) and repetitive (four or more yearly episodes) NSSI and concluded that four or more episodes per year would be a meaningful cut-off point, and the association was stronger between repetitive NSSI and suicidality. In a later study, Brunner and colleagues [42] proposed a *lifetime* prevalence of five or more NSSI episodes as a cut-off for repetitive NSSI. In the proposed DSM-5 NSSI disorder [43], engagement in NSSI on five or more days during the last year has been suggested as one of several criteria identifying a more severely affected group. This suggested cut-off has, however, been questioned for being too inclusive [44].

#### Bottom-up approaches

Furthermore, there have been some initiatives to use bottom-up, data driven methods to categorize groups of NSSI. Klonsky and Olino [34] performed a latent class analysis, identifying four subgroups within a sample of adult participants with NSSI. The subgroups were characterized in order of increasing severity, as an “experimental” group, a “mild” group, a “multiple functions/anxious” group and an “automatic functions/suicidal” group, and the authors found that the functions of the NSSI behavior varied considerably between the groups. The authors concluded that while NSSI is often accompanied by psychiatric distress, this is not always the case and recommended careful clinical assessment when treating adolescents with a history of NSSI.

In a longitudinal study of NSSI, Moran et al. [45] found that, in contrast with the general sharp decrease in NSSI behavior around 16 years of age, a small share of individuals continued their NSSI going into early adulthood. Risk factors for belonging to the group that continued with NSSI included female sex and symptoms of depression and anxiety during adolescence. Tilton-Weaver [46] further identified three separate trajectories of NSSI between 13 and 17 years of age, one with NSSI peaking around the age of 15 and then declining, a second escalating throughout the teenage years and a third trajectory on a low but steady level.

The heterogeneity of the NSSI population was highlighted In a study by Stanford et al. [47] in 2018. The authors employed cluster analysis in a community sample of high school students with a history of NSSI and identified six psychological profiles: psychologically healthy, low impulsivity, poor coping, anxiety, impulsive, and pathological.

Furthermore, Uh et al. [48] used a clustering algorithm on a longitudinal dataset and identified two groups in young people with NSSI, one more severe “psychopathology” group and one less severe. The authors found that parental reports of the children’s emotional and behavioral development at a younger age could be used to predict membership of either group at age 14.

As is evident from the studies presented above, there is in the research field a growing interest in identification of subgroups among individuals engaging in NSSI. Several authors point out that knowledge of risk factors and predictors associated with different NSSI subgroups and trajectories is crucial to the planning of treatment and prevention strategies [46–48].

### The present study

Our intention in the present study is to follow up on some of the questions formulated by Robinson and Wilson [38], and thus adding to the body of knowledge regarding the characteristics of which groups can be identified in adolescents using different methods. Further, we intend to add to the few studies that have used exploratory, data-driven stratification in a sample of participants with NSSI [34, 47, 48].

In the first stage, we aim to investigate which groups indicate life-time prevalence (i.e., ever having engaged in the behavior) of NSSI on a single item versus a behavioral checklist but not on the single item (i.e., discrepant responders) in the same sample of Swedish community adolescents. Detailed statistics on a range of measures will be presented to facilitate comparison between the groups and shed light on commonalities and differences. Then, in a second stage, we will use exploratory data-driven analysis to identify groups in the participants indicating lifetime NSSI prevalence. Lastly, we aim to compare findings from the first and second stage and investigate to what degree identified groups map onto or overlap with each other. Our research questions are:

1. Which groups of adolescents with NSSI can be identified in the same sample by the single-item question and the behavioral checklist, and what are their characteristics?
2. Which groups can, in turn, be identified using cluster analysis?
3. To what degree do groups identified by cluster analysis map onto or overlap with the groups identified by single-item and behavioral checklist?

## Methods

### Procedure

The data in the present study were collected as part of a larger, longitudinal project aiming at evaluating preventive interventions targeting NSSI and mental health in junior high schools. First, project staff reached out to principals at lower secondary schools in Östergötland county, Sweden, informing about the project and inviting the schools to participate. Next, the caregivers of adolescents in the schools that were participating in the project were contacted with written information and informed consent forms for their child’s participation. Adolescents received oral and written information and signed an informed consent form after their caregivers had given their consent. Written informed consent from the adolescents and both of the adolescents’ caregivers (except in cases with only a single caregiver) was required for participation. Adolescents who did not want to participate had the option not to do so, even if their caregivers had given their consent.

The first wave of measurements, from which the data in the present study are taken, was carried out in January to March 2022. Students answered survey questions using their school computers/notebooks by means of a digital survey tool (REDCap) [49]. The participants filled out the surveys at school, during scheduled time, with study staff present to be able to follow up on any questions or reactions. School health staff (nurses, counselors) were informed beforehand and were available should their services be needed in connection with the data collection.

The study was approved by the Swedish Ethical Review Authority (2021 − 01699, 2021–05049).

### Participants

#### Participating schools

Out of 28 schools contacted by project staff, six were positive to participate. One additional school contacted study staff with an interest in participating and was included as well. In one of the schools, only about 5% of caregivers gave consent for their child to participate, so it was agreed not to proceed with data collection at that school, resulting in six schools. Five participating schools were administered by the local municipality and one privately.

Out of a total of 1,054 eligible students in the seventh and eighth grades in the six schools, a total of 266 (25.3%) adolescents agreed to participate and received written consent from both caregivers, and completed the first survey wave at their respective schools. Eligibility criteria for the study were enrollment in a regular school class, grade seven or eight, following the standard Swedish curriculum. Two participating schools belonged to a medium-sized city municipality, two to smaller city municipalities, and two to rural municipalities. Five schools were medium-sized (120–238 students in 7th and 8th grade), and according to publicly available demographics (SALSA) [50], had a somewhat lower proportion of students with a foreign background (6–22%, mean for all of Sweden is 27%) and around average higher proportion of students whose parents had received education beyond secondary level (45–63%, Swedish mean is 62%). The sixth school, located within a medium-sized city, was smaller in size (< 100 students in 7th and 8th grade), and had a higher proportion of students with a foreign background (> 50%). The proportion of girls was similar in all participating schools (41–49%).

#### Study sample

The study sample consisted of 266 adolescents aged 13–15 years (*M* = 14.21, *SD* = 0.56). In the sample, 155 (58.3%) identified as girls, 105 (39.5%) as boys and 6 (2.3%) as non-binary. Of the participants, 71.8% reported living with both parents (see Table 1).

**Table 1:** Participant demographics, *N* = 266

Characteristics	*n* (%)
Age, years (*M*, *SD*)	14.25 (0.56)
Gender	
Girls	155 (58.3)
Boys	105 (39.5)
Non-binary	6 (2.3)
Living with both parents	191 (71.8)
Region of origin	
Born in Sweden	253 (95.1)
Born in another European country	7 (2.6)
Born outside of Europe	6 (2.3)

### Measures

#### Demographic information

Questions regarding general background, such as gender, age, country of birth, parents’ work and housing arrangements, as well as questions about receiving help or support from mental health professionals were developed for the purpose of this study.

#### Self-injury

In order to measure multiple aspects of NSSI, several measures were included in the survey material. The single-item NSSI question “Have you ever actually engaged in purposely hurting yourself without wanting to die? (that is, intentionally hurting yourself without wanting to die? For example, cutting or burning yourself.)”, which participants answered by checking a box for yes or no, and items assessing suicide ideation (“Have you ever had thoughts of killing yourself?”) and attempt (“Have you ever made an actual attempt to kill yourself in which you had at least some intent to die?”)were included from the SITBI (short form, self-report, SITBI-SF-SR) [35]. The survey material also included the NSSI behavioral checklist from the ISAS [51], presented after the single-item question. The item ”swallowing dangerous substances” was excluded as it is not usually included in the definition of NSSI. In addition to lifetime prevalence, in order collect data on more recent NSSI frequency, an item for last year prevalence was included formulated by the research group in accordance with the DSM-5 definition, as well as an item for three-month prevalence. Dichotomous variables indicating whether participants had engaged in NSSI at least five times during the last year, as well as whether a participant had used at least five different NSSI methods, were computed. The first of these corresponds to Criterion A of the proposed NSSI disorder diagnosis (although the criterion refers to days instead of episodes) [52], while the second has been used previously as a cut-off for greater NSSI versatility [53]. Additionally, cognitions associated with NSSI were investigated through the Non-Suicidal Self-Injury Expectancy Questionnaire (NEQ) [18]. The original NEQ utilizes 25 items to measure five domains of NSSI expectations (affect regulation, negative social outcomes, communication, pain and negative self-beliefs), and participants rate expected outcomes on a five-point Likert scale ranging from 1 (“not likely at all”) to 5 (“extremely likely”). Here, in order to keep the survey within reasonable size, the two strongest indicators per factor, as demonstrated by Hasking and Boyes [18], were included.

#### Emotion regulation

Difficulties with emotion regulation were measured in the study sample using the Brief version of the Difficulties in Emotion Regulation Scale (DERS-16) [54], a shorter version of the original 36-item scale [55]. Participants rate items on a 5-point Likert scale ranging from 1 (“almost never”) to 5 (“almost always”). DERS-16 yields a total score as well as five sub-factor scores (Nonacceptance of emotional responses, Difficulties engaging in goal-directed behavior, Impulse control difficulties, Limited access to emotion regulation strategies and Lack of emotional clarity), where a higher score reflects greater emotion regulation difficulties. The scale has been validated multiple times in Swedish samples [54, 56].

#### Quality of life, subjective health and well-being

Quality of life, subjective health, and well-being were measured using the KIDSCREEN-52 questionnaire [57]. It consists of 52 items intended to measure health-related quality of life (HRQoL) across ten domains where higher scores reflect better HRQoL: physical well-being, psychological well-being, moods and emotions, self-perception, autonomy, parent relation and home life, financial resources, peers and social support, school environment and bullying. Participants answer, indicating either frequency (from “never” to “always”) or attitude (from “not at all” to “extremely”) on a five-point scale.

#### Stigmatization

Stigmatization was assessed using the Peer Mental Health Stigmatization Scale-Revised (PMHSS-R) [58, 59]. The PMHSS-R includes eleven statements concerning youth and mental health, and participants indicate the degree to which they agree on a Likert-type scale from 1 (“disagree completely”) to 5 (“agree completely”). The measure yields two subscale scores, one for Stigma awareness, related to the perception of stigma in general, and the other for Stigma agreement reflecting the degree to which participants’ own opinions are negative towards mental illness.

#### Self-criticism

To measure Self-criticism, the Self-Rating Scale (SRS) [60] was used. Participants rate the degree to which they agree with eight statements on a scale between 0 and 7. The scale yields a total score for the degree of reported self-criticism.

#### Help-seeking

Help-seeking was measured using instruments developed by Schmeelk-Cone et al. [61] with ten survey items generating three different scales. Participants indicate their answers on a scale between 1 (“strongly disagree”) to 4 (“strongly agree”). *Help-Seeking Acceptability (HSA)* aims at capturing respondents’ expectations associated with receiving help and being supported in seeking help. *Adult Help for Suicidal Youth (AHSY)* measures the experienced availability of adult support concerning suicidality and *Reject Codes of Silence (RCS)* measures subjective barriers to help-seeking in case of suicidality. Higher scores reflect more positive attitudes towards support, help-seeking and disclosure.

Data on estimated reliability (Cronbach’s alpha and McDonald’s Omega total) for all measures in the present sample are available as Supplementary Materials to the manuscript.

### Statistical analysis

Participants were categorized into groups so that one group consisted of participants indicating experience of NSSI on the single item (“single-item group”), and another group indicating NSSI on a behavioral checklist (but without a positive answer on the single item, thus with a discrepant pattern; “behavioral checklist group”), and a third group not indicating NSSI on any of the two measures. Descriptive statistics were presented with frequencies and percentages for categorical data, and means and standard deviations for continuous data, and calculated separately for the single-item group and the behavioral checklist group. Comparisons between the two groups were carried out using independent-samples t-test for continuous measures and, in the case of categorical measures, chi-square test. Effect sizes were calculated using Cohen’s *d* and Cramér’s *V*. For NSSI behaviors, odds ratios for belonging to the single-item group rather than the behavioral checklist group were calculated. In order to determine whether the no NSSI group, single-item group and behavioral checklist group were different in the domain of NSSI attitudes and expectations, one-way analysis of variance (ANOVA) and Bonferroni-corrected post-hoc t-tests were used.

Finally, data from the participants with NSSI, both the single-item group and the checklist group, were processed using hierarchical, agglomerative cluster analysis. The intention was to use a data-driven method of analysis to identify clusters within the NSSI subset, something which in agglomerative, hierarchical cluster analysis is achieved using a procedure where all data points are initially considered their own cluster and then in a step-by-step fashion combined into larger clusters. The variables used as basis for clustering, forming a four-dimensional Euclidean distance matrix, were difficulties with emotion regulation (DERS-16 total score), NSSI versatility (number of methods used), self-criticism (SRS total score) and Health-related Quality of Life (KIDSCREEN-52 total score). The agglomerative clustering method used was complete (maximum) linkage clustering, meaning that the procedure took into account all dissimilarities between two clusters and used the largest dissimilarity to calculate the distance between them. It has been shown that complete linkage clustering tends to produce clusters of data points that are close to each other in the n-dimensional space produced by the input variables, which suited our interests. Data included in the cluster analysis was inspected visually to check for outliers. The number of clusters to include was determined using the elbow method, increasing the number of clusters until further additions do not lead to a lower within-cluster variation (within-cluster sum of squares; WSS), while keeping real-world utility and meaning in mind.

Statistical analyses were carried out using the R software package (version 4.3.1) [62] and RStudio IDE (version 2023.09.0 + 463) [63], odds ratios calculated using the oddsratio() function from the epitools R package (version 0.5–10.1), and cluster analysis was performed using the hclust() function from the stats R package (version 4.3.1).

## Results

### NSSI Prevalence

Of the total sample of 266, 118 participants (44.4%) acknowledged NSSI at least once. In the investigated sample, the mean age of reported onset of NSSI (first episode) was 11.62 years (*SD* = 2.41). The range of the lower quartile was 2 to 10.75 years.

Of the 118 participants that acknowledged lifetime prevalence of NSSI, 55 (20.7% of the whole sample) did so on the single NSSI item. All but one of the participants in this group also specified at least one NSSI method on the behavioral checklist.

Another 63 participants (23.7% of the whole sample) indicated having engaged in at least one NSSI method on the behavioral checklist, even though they denied ever having engaged in NSSI on the single item.

### Characteristics of single-item and behavioral checklist groups

The single-item group (*n* = 55) consisted of 78.2% girls, 10.9% boys and 10.9% identified as non-binary. The behavioral checklist group (*n* = 63) consisted of 65.1% girls and 34.9% boys. Chi-square test showed these differences to be significant (χ^2^(2) = 14.7, *p* < 0.001, Cramér’s *V* = 0.35).

Overall, 30.8% of participants (including those not indicating a history of NSSI on any measure) reported ever having had suicidal thoughts and 7.5% reported ever having made a suicide attempt with at least some intent to die. Both self-reported suicidal thoughts and attempts were more commonly reported in the single-item group. See Table 2.

**Table 2:** Characteristics of NSSI groups

	Single-item (*n* = 55) *n* (%)	Behavioral checklist (*n* = 63) *n* (%)	Statistic
Gender			
Girls	43 (78.2%)	41 (65.1%)	χ2(2) = 14.72 *p* < 0.001 Cramér’s *V* = 0.35
Boys	6 (10.9%)	22 (34.9%)	
Non-binary	6 (10.9%)	0 (0%)	
NSSI			
Age of onset, years *M* (*SD*)	11.83 (2.31)	11.16 (2.55)	*t*(51.8) = -1.15 *p* = 0.26
One or more NSSI episode during last year	43 (78.2%)	23 (36.5%)	χ2(1) = 19.03 *p* < 0.001 Cramér’s *V* = 0.42
Five or more NSSI episodes during last year^a^	35 (63.6%)	14 (22.2%)	χ2(1) = 19.07 *p* < 0.001 Cramér’s *V* = 0.42
Number of NSSI methods *M* (*SD*)	6.22 (2.29)	3.32 (2.26)	*t*(113.5) = -6.90 *p* < 0.001 *d* = 1.27
Five or more NSSI methods^b^	44 (80%)	16 (25.4%)	χ2(1) = 32.88 *p* < 0.001 Cramér’s *V* = 0.54
Ever published their NSSI on social media	4 (7.3%)	0 (0%)	χ2 (1) = 1.76 *p* = 0.18 Cramér’s *V* = 0.19
Suicidality			
Suicidal ideation	42 (76.4%)	18 (28.6%)	χ2(1) = 24.96 *p* < 0.001 Cramér’s *V* = 0.48
Suicide attempt	16 (29.1%)	2 (3.2%)	χ2(1) = 13.32 *p* < 0.001 Cramér’s *V* = 0.36
NSSI disclosure and help-seeking			
Disclosure to family	16 (29.1%)	3 (4.8%)	χ2(1) = 11.13 *p* < 0.001 Cramér’s *V* = 0.33
Disclosure to friend(s)	28 (50.9%)	8 (12.7%)	χ2(1) = 18.46 *p* < 0.001 Cramér’s *V* = 0.41
Disclosure to a professional	12 (21.8%)	0 (0%)	χ2(1) = 13.01 *p* < 0.001 Cramér’s *V* = 0.36
Received professional mental health care/counseling	28 (50.9%)	16 (25.4%)	χ2(1) = 7.12 *p* < 0.01 Cramér’s *V* = 0.26
Received professional help regarding NSSI	14 (25.5%)	3 (4.8%)	χ2(1) = 4.71 *p* < 0.05 Cramér’s *V* = 0.25
Emotion regulation and self-criticism			
DERS-16 *M* (*SD*)	51.53 (14.72)	39.54 (14.32)	*t*(112.9) = -4.47 *p* < 0.001 *d* = 0.83
SRS *M* (*SD*)	34.98 (10.9)	24.83 (9.19)	*t*(106.2) = -5.43 *p* < 0.001 *d* = 1.01
Quality of life			
KIDSCREEN-52 *M* (*SD*)	150.26 (25.31)	175.08 (26.67)	*t*(113.8) = 5.16 *p* < 0.001 *d* = 0.95

*Note*. NSSI = Nonsuicidal self-injury. DERS-16 = Difficulties in Emotion Regulation Scale– short version (Higher scores reflect greater difficulties) [54]; SRS = Self-Rating-Scale (Higher scores reflect larger degree of self-criticism) [60]. *d* = Cohen’s *d*. ^a^ corresponding to Criterion A of the proposed NSSI disorder diagnosis. ^b^ used previously as a cut-off for greater NSSI versatility

Participants in the single-item group were more likely to have injured themselves intentionally during the last year than the behavioral checklist group (χ2(1) = 19.03, *p* < 0.001, Cramér’s *V* = 0.42) and were more likely to have had five or more NSSI episodes during the last year (χ2(1) = 32.88, *p* < 0.001, Cramér’s *V* = 0.54). A larger share of the participants in the single-item group also reported having disclosed their NSSI to someone else (to family, χ2(1) = 11.13, *p* < 0.001, Cramér’s *V* = 0.33; to friend(s), χ2(1) = 18.46, *p* < 0.001, Cramér’s *V* = 0.41; to a professional, χ2(1) = 13.01, *p* < 0.001, Cramér’s *V* = 0.36). The single-item group had higher mean scores on measures of difficulties with emotion regulation, DERS-16 (*t*(112.9) = -4.47, *p* < 0.001, *d* = 0.83), and self-criticism, SRS (*t*(106.2) = -5.43, *p <* 0.001, *d* = 1.01), indicating a larger burden in these domains, and also had a higher mean number of NSSI methods (*t*(113.5) = -6.90, *p* < 0.001, *d* = 1.27). Further, the single-item group had significantly lower mean score on the health-related quality of life measure, KIDSCREEN-52 (*t*(113.8) = 5.16, *p* < 0.001, *d* = 0.95). See Table 2. Reported NSSI episodes, for both groups combined, during the last three months ranged between one and 100 episodes, with a mean of 12 and a median of 5. For NSSI episodes during the last year, the range was 1–200, with a mean of 35 and a median of 6.

Data on NSSI frequency during the last year, as well as the last three months, indicated that while some of the participants engaged in NSSI on a more than weekly basis, several participants seemingly had not established NSSI as a behavioral pattern and whose NSSI episodes were occasional (number of episodes last three months: range 1-100, *M* = 11.71, *SD* = 18.15, mode = 1; last year: range = 1-200, *M* = 34.76, *SD* = 50.44, mode = 1).

**Fig. 1:**
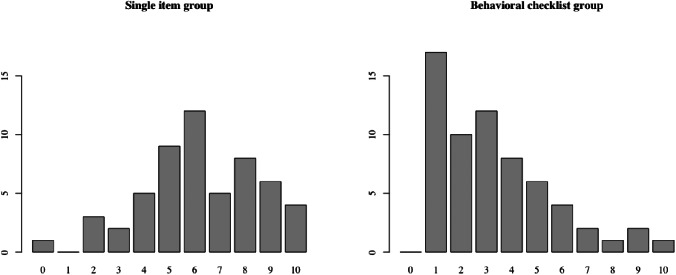
Number of NSSI methods per participant. *Note.* NSSI: Nonsuicidal self-injury

The validity of NSSI method versatility (i.e., number of NSSI methods that participants reported having used) as an indicator of severity was evaluated in the present sample by calculating Pearson’s product-moment correlations with difficulties with emotion regulation (DERS-16; *r* = 0.59, *p* < 0.001), self-criticism (SRS; *r* = 0.52, *p* < 0.001) and quality of life, subjective health and well-being (KIDSCREEN-52; *r* = -0.56, *p* < 0.001). Methods of NSSI were also compared between the single-item group and the behavioral checklist group. The single-item group had a significantly higher versatility, having used a mean of 6.22 methods (*SD* = 2.29) while the behavioral checklist group had used a mean of 3.22 methods (*SD* = 2.26; two-tailed t-test *t(113.5)* = 6.90, *p* < 0.001, *d* = 1.27; see Fig. 1). Further, there were patterns regarding which methods were more and less common. Comparison of relative frequencies of the different methods between the single item and the behavioral checklist groups revealed that some methods were equally common in both groups. This applies to pinching and sticking oneself as well as interfering with wound healing, methods that were about as common in the behavioral checklist group as in the single-item group (*OR* of belonging to the single-item group provided pinching or sticking oneself was 1.53 and 1.80 respectively, and 1.75 for interfering with wound healing), while some methods were several times more common in the single-item group (severe scratching, carving, burning and cutting, with *OR*s of 5.18, 7.67, 7.73 and 27.82, respectively, see Table 3.

**Table 3:** NSSI methods in single item and behavioral checklist groups

	Single-item *n* (%)	Behavioral checklist *n* (%)	Odds Ratio (95% CI)
Cutting	41 (74.55%)	6 (9.52%)	27.82 (9.86–78.49)
Burning	22 (40%)	5 (7.94%)	7.73 (2.68–22.34)
Carving	29 (52.73%)	8 (12.7%)	7.67 (3.08–19.07)
Severe scratching	38 (69.09%)	19 (30.16%)	5.18 (2.36–11.35)
Rubbing skin against rough surface	23 (41.82%)	10 (15.87%)	3.81 (1.61–9.02)
Biting oneself	41 (74.55%)	28 (44.44%)	3.66 (1.67–8.02)
Banging or hitting self	41 (74.55%)	33 (52.38%)	2.66 (1.22–5.82)
Sticking self with needles	27 (49.09%)	22 (34.92%)	1.80 (0.86–3.77)
Interfering with wound healing	40 (72.73%)	38 (60.32%)	1.75 (0.81–3.82)
Pinching	40 (72.73%)	40 (63.49%)	1.53 (0.70–3.36)

*Note*. NSSI = Nonsuicidal self-injury. Percentages of the participants who indicated having used the methods specified. The instrument allows indicating multiple methods. Odds Ratio of being part of the single-item group for participants responding positively on the item in question

Upon examination of differences between the single-item, the behavioral checklist and the no NSSI group on measures in the attitudes and expectations domain, some patterns emerged. Statistics are presented in Table 4. There was a significant difference regarding the expectations associated with the emotion regulation function of NSSI, in that the single-item group reported higher emotion regulation expectations, while the behavioral checklist group was similar to the no NSSI group. The present sample data did not indicate differences between the groups’ expectations regarding pain, negative social outcomes, using NSSI as a means of communication or effects on their self-image. Further, the single-item group seemed to be aware of stigmatization of mental suffering to a significantly larger degree than the group without NSSI. For help-seeking, analysis of variance and post hoc comparison indicated differences between the groups on all three subscales. The single-item group scores were significantly lower than those from the no NSSI group on all three subscales, indicating that they were less prone to seek help. They also differed significantly from the no NSSI group (but not from the behavioral checklist group) on the RCS scale, indicating that they reported greater subjective barriers toward help-seeking in case of suicidal ideation. The behavioral checklist group had a significantly lower score than the no NSSI group on the Adult Help for Suicidal Youth (AHSY) subscale.

A large proportion of participants (81.8%) that reported having engaged in NSSI on the single-item also reported that they wanted to stop injuring themselves, while the equivalent proportion in the checklist group was 60.3%. A bit over two-thirds of the single-item group further reported that they had told someone else about injuring themselves, something that was reported by around one fifth of the participants in the behavioral checklist group. Half of the single-item group (50.9%, compared to 24.8% in the whole sample) reported having received professional mental health and little over one in four (25.5%) had received help specifically for NSSI. In the checklist group, these proportions were 25.4% and 4.8%.

**Table 4:** NSSI attitudes and expectations in the identified groups with and without NSSI, means and standard deviations

	No NSSI (I) (undefined *M*, undefined *SD*)	Single-item (II) (undefined *M*, undefined *SD*)	Behavioral checklist (III) (undefined *M*, undefined *SD*)	Statistics	Post hoc comparisons
NEQ Affect regulation	3.18 (1.38)	5.42 (1.79)	3.59 (1.23)	*F*(2, 263) = 49 *p* < 0.001 η^2^ = 0.269	I-II *p* < 0.001 I-III *p* = 0.202 II-III *p* < 0.001
NEQ Negative social outcomes	4.83 (1.72)	4.62 (1.52)	4.98 (1.62)	*F*(2, 263) = 0.691 *p* = 0.502	
NEQ Communication	3.22 (1.36)	3.16 (1.51)	3.28 (1.51)	*F*(2, 263) = 0.088 *p* = 0.916	
NEQ Pain	6.19 (1.88)	6.2 (1.73)	6.41 (1.69)	*F*(2, 263) = 0.341 *p* = 0.712	
NEQ Negative self-beliefs	5.38 (1.73)	5.84 (1.61)	5.76 (1.6)	*F*(2, 263) = 2.036 *p* = 0.133	
Stigma awareness	14.45 (5.04)	16.73 (5.55)	15.59 (4.79)	*F*(2, 262) = 4.26 *p* = 0.015 η^2^ = 0.033	I-II *p* = 0.015 I-III *p* = 0.454 II-III *p* = 0.706
Stigma agreement	7.41 (2.93)	7.27 (3.25)	7.07 (2.9)	*F*(2, 262) = 0.274 *p* = 0.761	
HSA	11.39 (2.89)	10.27 (2.32)	10.52 (3.04)	*F*(2, 262) = 4.1 *p* = 0.018 η^2^ = 0.025	I-II *p* = 0.038 I-III *p* = 0.139 II-III *p* = 1
AHSY	9.39 (1.99)	8.16 (2.11)	8.57 (2.17)	*F*(2, 262) = 8.599 *p* < 0.001 η^2^ = 0.06	I-II *p* < 0.001 I-III *p* = 0.029 II-III *p* = 0.885
RCS	9.3 (1.84)	8.27 (2.16)	8.81 (1.95)	*F*(2, 262) = 5.909 *p* = 0.003 η^2^ = 0.045	I-II *p* = 0.003 I-III *p* = 0.317 II-III *p* = 0.425

*Note*. Means and, in parentheses, standard deviations of scores for the No NSSI (I, *N* = 153), Single-item (II, *N* = 55) and Behavioral checklist (III, *N* = 58) groups. NEQ: Nonsuicidal Self-injury Expectancy Questionnaire. HSA: Help Seeking from Adults in School. AHSY: Adult Help for Suicidal Youth. RCS: Reject Codes of Silence. On the NEQ subscales, higher scores indicate greater expectancy of the various effects of NSSI. On the Stigma subscales, higher scores indicate greater Stigma awareness and agreement. On the Help-seeking scales, higher scores indicate greater likelihood or lesser barriers to seeking help. Statistics from One-way ANOVA (outcome ~ group). Post hoc pairwise t-tests with Bonferroni correction

### Cluster analysis of adolescents with NSSI

In the last stage of analysis, identifying data-driven subgroups of adolescents with NSSI, hierarchical cluster analysis of the NSSI group was performed. Participants that reported NSSI on the single-item or the behavioral checklist were included, resulting in a total of 118 subjects. After processing of the distance matrix, a solution with two clusters was suggested to be the optimal one. Visual inspection of the cluster dendrogram (see Supplementary Materials) revealed that this would result in one small and one very large cluster, so it was decided to proceed to the next branching. The resulting division yielded three clusters containing 11, 40 and 67 participants, respectively.

**Fig. 2:**
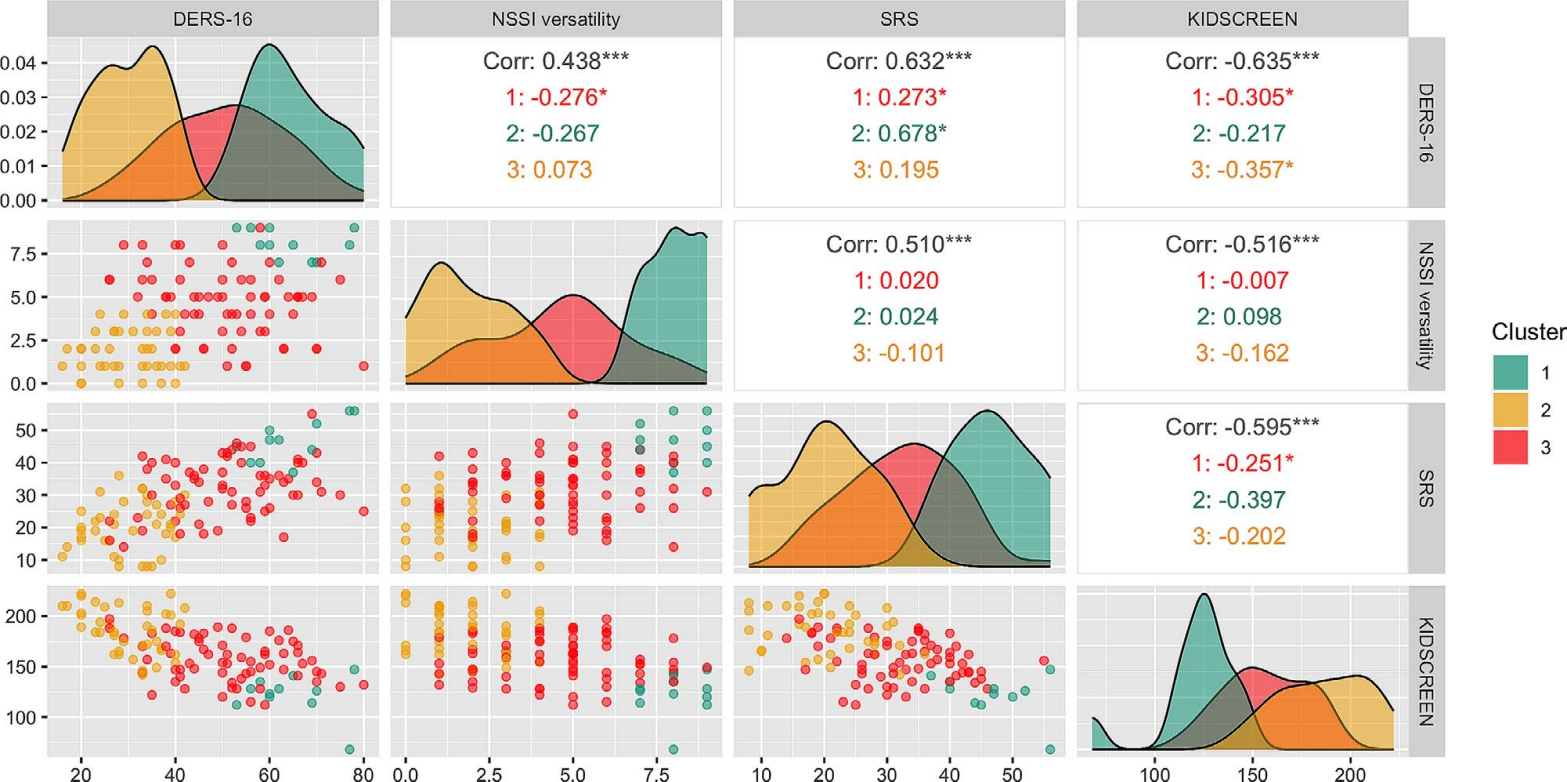
Cluster analysis visualization. *Note.* NSSI: Nonsuicidal self-injury. DERS-16: Difficulties in Emotion Regulation Scale– short version (Higher scores reflect greater difficulties) [54]; SRS, Self-Rating-Scale (Higher scores reflect larger degree of self-criticism) [60]

Descriptive statistics for all three clusters, are reported in Table 5, and the clusters are presented graphically in Fig. 2. The smallest cluster, Cluster 1 (*n* = 11), was the first to be separated in the analysis, and was the cluster most severely affected by NSSI and other symptoms. In this cluster, scores on difficulties with emotion regulation as well as self-criticism were high relative to the other clusters, as was self-reported suicidality, and the number of NSSI methods was almost at the maximum. The mean score on KIDSCREEN was lower, indicating a lower general health-related quality of life.

Of the remaining clusters, Cluster 2 (*n* = 40) was a seemingly less severely affected sub-population, hardly overlapping with the first severe group. Among participants in Cluster 2, only a minority indicated having engaged in NSSI on a single NSSI item, while a majority acknowledged behavioral checklist prevalence of NSSI. Further, the mean number of NSSI methods was low, as were scores on difficulties with emotion regulation and self-criticism, and the mean score on KIDSCREEN was higher. Cluster 3 was the largest (*n* = 67) and overlapped with the two others, as participants’ response patterns across measures were less coherent.

**Table 5:** Descriptive statistics for clusters

	Cluster 1 (*n* = 11)	Cluster 2 (*n* = 40)	Cluster 3 (*n* = 67)
Gender			
Girls, *n* (% of cluster)	6 (54.5)	24 (60.0)	54 (80.6)
Boys, *n* (% of cluster)	2 (18.2)	16 (40.0)	10 (14.9)
Non-binary, *n* (% of cluster)	3 (27.3)	0 (0.0)	3 (4.5)
NSSI			
Positive on NSSI single-item, *n* (% of cluster)	10 (90.91)	7 (17.50)	38 (56.72)
Positive (exclusively) on NSSI behavioral checklist, *n* (% of cluster)	1 (9.09)	33 (82.50)	29 (43.28)
Age at first NSSI episode, *M* (*SD*)	11.33 (2.09)	11.66 (2.59)	11.62 (2.44)
Number of NSSI methods, *M* (*SD*)	9.09 (0.83)	2.35 (1.35)	5.33 (2.17)
Five or more NSSI methods, *n* (% of cluster)	11 (100)	3 (7.5)	46 (68.66)
Number of NSSI episodes during the last year, *M* (*SD*)	46.86 (70.98)	14.23 (34.92)	40.76 (145.63)
Five or more NSSI episodes during last year, *n* (% of cluster)	7 (63.64)	9 (22.50)	33 (49.25)
Suicidality			
Suicidal ideation *n* (% of cluster)	11 (100.00)	8 (20.00)	41 (61.19)
Suicide attempt *n* (% of cluster)	9 (81.82)	1 (2.50)	8 (11.94)
Emotion regulation and self-criticism			
DERS-16, *M* (*SD*)	64.36 (8.26)	29.95 (7.33)	51.03 (12.45)
SRS, *M* (*SD*)	46.73 (6.37)	20.40 (7.6)	32.21 (8.66)
Quality of life			
KIDSCREEN-52 total, *M* (*SD*)	122.00 (20.8)	186.3 (22.58)	156.82 (21.15)

*Note*. NSSI = Nonsuicidal self-injury. DERS-16 = Difficulties in Emotion Regulation Scale– short version (Higher scores reflect greater difficulties) [54]; SRS = Self-Rating-Scale (Higher scores reflect larger degree of self-criticism) [60]; KIDSCREEN-52 (Higher scores reflect better health-related quality of life) [57]

## Discussion

One aim in the present study was to identify, describe and compare the groups among Swedish community adolescents who indicate NSSI on a single-item and on a behavioral checklist. We found that 20.7% of the participants in our sample acknowledged lifetime prevalence of NSSI when asked a general NSSI question with a single item: “Have you ever actually engaged in hurting yourself without suicidal intent (that is, purposely hurt yourself without wanting to die, for example by cutting or burning)?” [35]. An additional 23.7% indicated having engaged in NSSI using at least one method on a behavioral checklist and at the same time did not report NSSI experience on the single item, placing the total lifetime prevalence rate of NSSI at least once in the sample at 44.4%. Comparison data were presented for the single-item group and the behavioral checklist group. Results indicated significant differences in gender composition, emotion regulation, self-criticism, health-related quality of life, NSSI methods, frequency, versatility and attitudes toward help-seeking and disclosure between groups, with higher NSSI frequency and greater distress in the participants in the single-item group. The mean number of NSSI methods was 6.22 in the single-item group, and 3.22 in the behavioral checklist group. Lastly, the cluster analysis of the subset of participants with NSSI resulted in a solution with three clusters, two of which had little overlap with each other and to a certain degree matched the single-item and behavioral checklist groups.

### Groups identified by single-item and behavioral checklist measures

The finding that behavioral checklists yield around doubled prevalence estimates corroborates previously published results [2, 30, 36, 64] and shows that this pattern holds also in community samples of younger adolescents. Further, the single-item group had a significantly larger proportion of girls as well as higher mean NSSI frequency and versatility. The findings of Robinson and Wilson [38], were thus corroborated also in adolescents. Similarly, the finding that NSSI methods associated with greater severity [26] and fitting closer to NSSI stereotypes (i.e., being a girl or young woman and self-injuring by cutting) [65, 66] were more common in the single-item group confirm previous results. Robinson and Wilson [38] suggested that single NSSI survey items are sensitive to the degree to which a participant identifies as someone who self-injures and thus tend to select those closer to a perceived stereotype while excluding others, that is, participants might “evaluate their own behavioral history in light of their personal understanding of what self-injury is, and who self-injures” (p. 728) [38]. This hypothesis, previously also put forward by Swannell and colleagues [30], also holds in light of the results in the present study, as female gender and more stereotypical NSSI behaviors increased the likelihood for a participant to answer yes on the single NSSI item.

When the behavioral checklist group and the single-item group were compared on other variables such as self-reported suicide attempts, suicidal ideation, emotion regulation difficulties, self-criticism, NSSI versatility, and number of episodes during the last year, it became even more apparent that the behavioral checklist group *in general* was less severely affected than the single-item group. A higher proportion of participants in the behavioral checklist group answered that they had stopped hurting themselves and the proportion of participants reporting NSSI episodes in the last year was smaller. This was previously demonstrated by Lund et al. [37]. However, in the present sample, there was variability also within the behavioral checklist group. The rates for five or more NSSI methods as well as five or more episodes during the last year, cut-off levels potentially indicating moderate or severe NSSI, were both around 25% in the behavioral checklist group. Seemingly, a non-trivial share of individuals who engage in NSSI do so in a severe and repeated way without identifying as someone who self-injures. Thus, as it seems a single NSSI item is sensitive to participants’ degree of identification with a self-injury stereotype but not very specific when it comes to detection of severe or repetitive NSSI.

Age of onset did not differ significantly between the single-item and checklist group (*M* = 11.83 and 11.16), but is, all the same, worth commenting on. Age of onset has previously been observed to be around 12–14 years of age [7, 11, 12], and the somewhat lower age reported by adolescents in the current study in 2022 is disconcerting. This is something that needs to be monitored going forward, as it has implications for when and where preventive work might have the largest impact, for example.

In line with the results presented by Simone and Hamza [23], we found that participants in the single-item group were more likely to have talked to someone else about their NSSI. One third of the participants in the single-item group, and four out of five in the behavioral checklist group, had never disclosed their NSSI experiences. Comparison of the group scores on attitudes toward help-seeking revealed that both groups with NSSI experience were significantly more pessimistic concerning availability and effectiveness of support than the no NSSI group. The single-item group was also more inclined to answer that in case of suicidal ideation, adolescents who didn’t want help should be left alone. Echoing the findings of Rowe and colleagues [22], the results show that the adolescents in the present sample preferred to be open about their NSSI toward family and friends, when they decided to disclose their NSSI. Only a fraction of the individuals with NSSI reported having received treatment for NSSI specifically (25.5% and 4.8%, respectively, in the single-item and behavioral checklist groups), which is in line with earlier research [22], and thus unfortunately still seems to hold true for adolescents in 2022.

As far as we know, this study is the first to present results from the Nonsuicidal Self-Injury Expectancy Questionnaire [18] from a child or adolescent sample. The questionnaire was employed in a shortened form in the current study, for practical reasons, with two items per factor instead of five. Also, further studies on the performance of the NEQ are required in order to determine validity and psychometric properties in adolescent populations. Results tentatively support the notion that expectations of regulating emotions by NSSI are higher among individuals with more severe NSSI. This is in line with previous findings from deployment of the NEQ in adult populations (e.g. [20])., and also fits previous results (e.g. [5]). showing that emotion regulation is an important function of NSSI. On the other domains of the NEQ, data did not support differences between the groups. Findings of differences on the NEQ domains of Pain and Communication between subjects with and without NSSI, presented by Dawkins et al. [20], were thus not reproduced in the present study. Caution must be taken when interpretating results, however, due to the fact that only some of the items from NEQ were included in the present study.

### Groups identified by cluster analysis

A further aim of the present study was to, for the first time, employ cluster analysis to identify groups among those with a history of NSSI in a community sample of young adolescents. The analysis singled out a small cluster (Cluster 1, about 10% of the NSSI population in the sample) with repetitive NSSI. In this cluster, all participants acknowledged suicidal ideation and many reported having made a suicide attempt, with at least some intent to die. A large share of the participants indicated NSSI on the single-item which, as mentioned above, can be interpreted as identifying as someone engaging in self-injury. Emotion regulation difficulties were high, as was self-criticism and NSSI versatility. This cluster included individuals that displayed higher levels of severity than the other clusters on almost all variables. It overlaps or matches the “automatic functions/suicidal” group found by Klonsky and Olino [34], the “psychopathology” group from the study by [48] and the “pathological” group found by Stanford et al. [47]. To the best of our knowledge, this is the first time that results have been replicated in young adolescents.

Cluster 2 constituted about a third of the NSSI population in the sample, and was less burdened by symptoms, although still with experience of self-injury. Few individuals in this cluster acknowledged NSSI on a single item, suggesting that many of the participants in the group engaged in NSSI without having an identity as someone who self-injures. Cluster 2 matched both the “experimental” and the “mild” group from the study by Klonsky and Olino [34] and the “psychologically healthy” and “low impulsivity” groups from Stanford et al. [47]. Despite some less severe characteristics, it should be stressed that the existence and size of this group need attention as it has a potential heightened risk of further NSSI [8]. Furthermore, this identified group is important to acknowledge as its participants might be those who benefit the most from low-level, universal preventive measures, as argued by Stanford and colleagues [47], who analyzed risk behaviors and identified a similar group in their sample.

Third, there was a cluster that overlapped with the others in a way that neither the first nor the second group did. The participants in Cluster 3 are probably best defined as “in between”, not belonging to either of the others, and there was a greater variability in the scores grouped together in this cluster. This is expected, as Cluster 3 consisted of data remaining after the first two and more coherent clusters had been identified, and the cluster thus included participants with experience of NSSI not closely matching the profiles of the other clusters. In the study by Klonsky and Olino [34], the group placed in between the “experimental” or “mild” and the “automatic functions/suicidal” groups was labeled “multiple functions/anxious group”, and Cluster 3 in the present study potentially corresponds to that group as it is neither “mild” nor “severe”. Further, if our above suggestions of how Clusters 1 and 2 correspond to groups in the study by Stanford and colleagues [47] are correct, the last three groups (“anxiety”, “poor coping” and “impulsive”) might correspond to Cluster 3. As it seems, this is a cluster where some risk factors or problematic tendencies are manifested but not necessarily in multiple areas simultaneously. The plurality of possible mechanisms behind NSSI, and hence the necessity of differentiated treatment options is also stressed by [47]. A significant share (43.28%) of the adolescents in this group did not acknowledge NSSI on the single item. These are young adolescents who are still in early adolescence, and identifying and assessing need for support for the individuals in this group should be a priority, as they face a potential risk of further problematic development. These adolescents can potentially be the target group for indicative preventive measures as well as efforts to increase the availability of counseling and therapy in schools and primary care.

As presented in Table 5, the proportions of participants indicating having engaged in NSSI five times or more during the last year suggest that this cut-off would be a weak predictor for cluster membership (63.64%, 22.50% and 49.25% respectively in Cluster 1, 2 and 3). It thus seems that many adolescents engage in NSSI with a frequency of five or more episodes per year without belonging to the group with higher severity of NSSI. We therefore agree with the criticism put forward by Muehlenkamp et al. [44] that the proposed frequency criterion could be set too low.

Further, looking at how response patterns on the single-item and behavioral checklist vary across the three clusters, a large majority of participants in Cluster 1 indicate NSSI on the single-item. This pattern is reversed for participants in Cluster 2. Cluster 1 and Cluster 2 thus overlap to a large degree with the single-item group and the behavioral checklist group. Participants in Cluster 3, however, are evenly distributed across the two groups. This strengthens the finding that while it is the case that many individuals with severe NSSI acknowledge NSSI on a single-item, the reverse does not hold. Individuals indicating NSSI only on a behavioral checklist are not necessarily mildly affected but can belong to the in-between group in this study identified as Cluster 3.

### Limitations

The main limitation of this study is the small sample size, and the composition of it. It must be recognized that even though efforts were made to minimize sampling bias, the recruitment process could not completely avoid it. This is especially because active consent from both caregivers was necessary for adolescents to participate. Results from previous studies (e.g. [67]) indicate that survey non-participation, non-response and attrition affects disadvantaged groups to a larger degree. Participant demographics indicate that the participation rate might be lower among adolescents born outside of Sweden, but this cannot be determined as the statistics for the included schools only concern the parents’ background. Psychiatric conditions have also been shown to lead to increased non-participation and drop-out rates [68]. However, prevalence rates of NSSI found in the current study sample confirm rates in earlier studies of similar age groups in Sweden [69, 70], which potentially argues against a bias toward having recruited a less disadvantaged group. It should also be noted that the single-item NSSI question in our survey explicitly mentions cutting and burning as examples of NSSI methods. As these methods also turned out to be the ones most strongly associated with answering yes on said question, one might suspect that the mentioning of the methods amplifies the suggested effect of NSSI stereotypes further. However, previous studies have used items with more examples [38] and with no examples [37] and still found the same overall discrepancy between single-item and behavioral checklist measures, and the same methods to be the most commonly reported. Some of the instruments were also, for pragmatic reasons, included only in part in the survey material. Thus, inferences need to be made cautiously as the validity of those scales was examined in their full form. Other instruments are included in their short form-version, whereas the long form could have increased the sensitivity of the measurements and yielded better internal consistency (see Supplementary Materials).

### Recommendations and future directions

The results in the current study come with several recommendations. A way forward suggested by Robinson and Wilson [38] is to be more clear about the effects of the operationalization of NSSI when collecting survey or interview data and to differentiate between *behaviorally identified* and *self-identified* NSSI. When single-item is used, perhaps in the interest of survey length and participant compliance, researchers need to be transparent about the fact, now underlined also by the present study sample of adolescents, that the detected group must be assumed to be skewed toward those with a larger burden of distress and a self-injury identity closer to perceived NSSI stereotypes. In the same manner, studies employing only behavioral checklists can be expected to detect a larger and more varied group with NSSI experience, including more individuals who only rarely engaged in NSSI, and therefore need to be aware of the risk of potential overinclusion. Depending on the research questions, both methodological options could be feasible. When the aim is to estimate NSSI prevalence, we believe that behavioral checklists should be the preferred choice. If, on the other hand, the aim is to identify participants with more severe NSSI, single-item might be a practical solution.

When planning preventive work, survey data featuring NSSI behavioral checklists can provide a broader picture of the situation and is sensitive also to individuals with low-frequent and non-stereotypical NSSI behaviors. In clinical settings, the results in the present study point to the importance of asking patients about specific NSSI behaviors to avoid missing information that could be crucial when planning treatment. Our data imply that there are a nontrivial number of adolescents who harm themselves repeatedly without using any of the stereotypical methods.

The results in the present study also underline the considerable challenge faced by school health staff and mental health professionals. Despite the societal trend toward greater openness regarding mental health issues [71], adolescents with experience of NSSI are hesitant to disclose the behavior, and especially so when talking to professionals. Preventive interventions that successfully reduce stigma and shame associated with NSSI, as well as broaden the stereotypical image of self-injury, might prove to be a valuable step toward this mean. As many adolescents in the present study reported that they would preferably disclose NSSI to family or friends, efforts to strengthen these supporting networks, for example concerning how to respond when told about NSSI, could be beneficial.

To sum up, the present study contributes toward a better understanding of different subgroups and the diversity among community adolescents with experience of NSSI. In alignment with previous explorations outside of clinical settings, we identified groups that differ significantly from each other and therefore potentially need different prevention and treatment efforts going forward. We have also contributed to the understanding of what effects can be expected when designing survey or interview research studies featuring single NSSI items or behavioral checklists.

### Electronic supplementary material

Below is the link to the electronic supplementary material.

**Supplementary Material 1:** Reliability estimates and cluster dendrogram

## Data Availability

The datasets generated and analyzed during the current study are not publicly available due to limitations written into the participants’ consent forms. Group level data are available from the corresponding author on reasonable request.

## Abbreviations

AHSY: Adult Help for Suicidal Youth
ANOVA: Analysis of Variance
DERS-16: Brief Version of the Difficulties in Emotion Regulation Scale
HRQoL: Health-Related Quality of Life
HSA: Help-Seeking Acceptability
ISAS: Inventory of Statements about Self-Injury
NSSI: Nonsuicidal Self-Injury
NEQ: Non-Suicidal Self-injury Expectancy Questionnaire
PMHSS-R: Peer Mental Health Stigmatization Scale-Revised
RCS: Reject Codes of Silence
SITBI-SF-SR: Self-Injurious Thoughts and Behaviors Questionnaire, Short-Form Self-Report
SRS: Self-Rating Scale
