# Supplementary material for: Measurement and stratification of nonsuicidal self-injury in adolescents
Source: BMC Psychiatry. 2024 Feb 7;24:107. doi: 10.1186/s12888-024-05535-3 (PMC10848387; doi:10.1186/s12888-024-05535-3)

**Supplementary materials**

Erik Aspeqvist, Linköping university
erik.aspeqvist@liu.se

1. **Reliability estimates for scales and subscales**

| Scale | Cronbach’s α | McDonald’s ω_t_ |
| --- | --- | --- |
| DERS-16 (five factors) | 0.95 | 0.97 |
| SRS | 0.88 | 0.92 |
| KIDSCREEN-52 Physical Well-Being | 0.83 | 0.88 |
| KIDSCREEN-52 Psychological Well-Being | 0.93 | 0.95 |
| KIDSCREEN-52 Moods and Emotions | 0.93 | 0.96 |
| KIDSCREEN-52 Self Perception | 0.89 | 0.92 |
| KIDSCREEN-52 Autonomy | 0.85 | 0.87 |
| KIDSCREEN-52 Parent Relations and Home Life | 0.91 | 0.93 |
| KIDSCREEN-52 Financial Resources | 0.86 | 0.87 |
| KIDSCREEN-52 Peers and Social Support | 0.85 | 0.93 |
| KIDSCREEN-52 School Environment | 0.88 | 0.93 |
| KIDSCREEN-52 Bullying | 0.72 | 0.76 |
| KIDSCREEN-52 Total (ten factors) | 0.97 | 0.98 |
| PMHSS-R Awareness | 0.83 | 0.88 |
| PMHSS-R Agreement | 0.80 | 0.85 |
| PMHSS-R Total (two factors) | 0.84 | 0.88 |
| Help-seeking HAS | 0.80 | 0.88 |
| Help-seeking AHSY | 0.76 | 0.77 |
| Help-seeking RCS | 0.62 | 0.72 |
| Help-seeking Total (three factors) | 0.83 | 0.89 |
| NEQ Affect regulation | 0.70 | NA* |
| NEQ Negative social outcomes | 0.41 | NA* |
| NEQ Communication | 0.66 | NA* |
| NEQ Pain | 0.75 | NA* |
| NEQ Negative self-beliefs | 0.57 | NA* |
| NEQ Total (five factors) | 0.64 | 0.81 |

*Note*. *: the number of items per subscale was too small to calculate ω_t_.

1. **Cluster dendrogram**


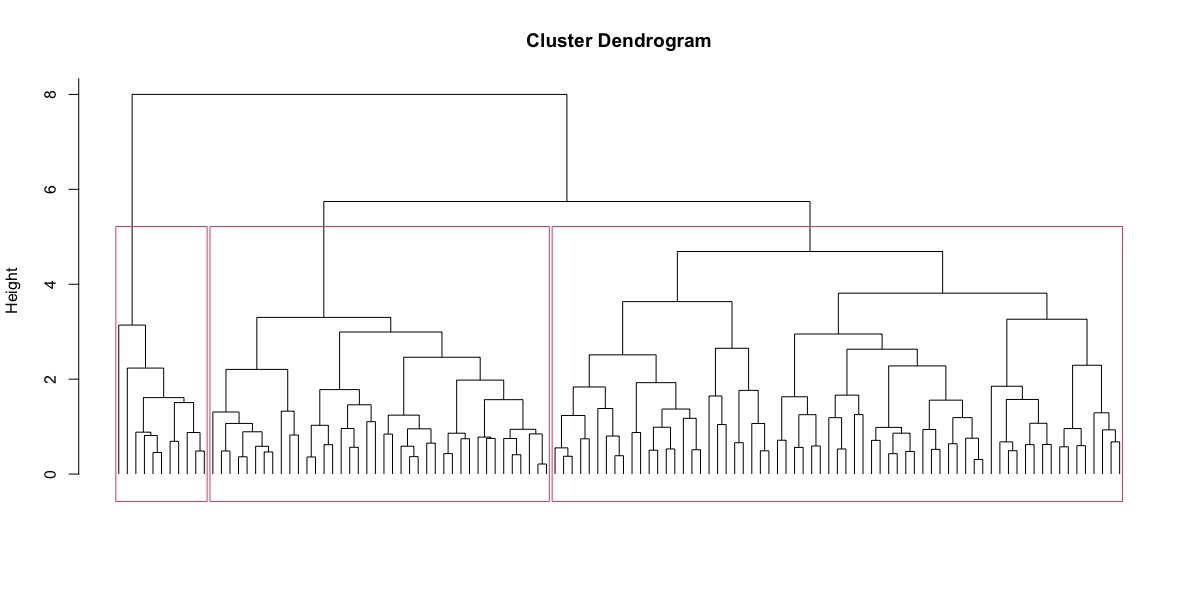

Supplement: Supplementary file 1 — Supplementary Material 1: Reliability estimates and cluster dendrogram [file 12888_2024_5535_MOESM1_ESM.docx]
